# 2,2′-[1,3-Diazinane-1,3-diylbis(methyl­ene)]bis­(4-bromo­phenol)

**DOI:** 10.1107/S1600536812001985

**Published:** 2012-01-21

**Authors:** Augusto Rivera, Ginna Paola Trujillo, Jaime Ríos-Motta, Karla Fejfarová, Michal Dušek

**Affiliations:** a Departamento de Química, Universidad Nacional de Colombia, Ciudad Universitaria, Bogotá, Colombia; b Institute of Physics ASCR, v.v.i., Na Slovance 2, 182 21 Praha 8, Czech Republic

## Abstract

The title compound, C_18_H_20_Br_2_N_2_O_2_, the heterocyclic ring adopts a chair conformation. The benzene rings make dihedral angles of 86.84 (10) and 60.73 (10)° with the mean plane of the heterocyclic ring. The dihedral angle between the two benzene rings is 79.77 (10)°. The mol­ecular structure is stabilized by two intra­molecular hydrogen bonds between the phenolic hy­droxy groups and N atoms with graph-set motif *S*(6). The crystal structure is stabilized by weak C—H⋯π inter­actions.

## Related literature

For related structures, see: Rivera *et al.* (2012 ▶, 2011 ▶). For the synthesis of the precursor, see: Rivera *et al.* (2010 ▶). For bond-length data, see: Allen *et al.* (1987 ▶). For Cremer–Pople puckering parameters, see: Cremer & Pople (1975 ▶). For graph-set notation, see: Bernstein *et al.* (1995 ▶). 
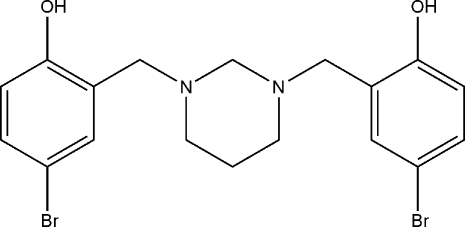


## Experimental

### 

#### Crystal data

C_18_H_20_Br_2_N_2_O_2_
*M* *_r_* = 456.2
Orthorhombic, 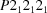

*a* = 5.9602 (3) Å
*b* = 17.2164 (8) Å
*c* = 17.7222 (8) Å
*V* = 1818.53 (15) Å^3^
*Z* = 4
Cu *K*α radiation
μ = 5.76 mm^−1^
*T* = 120 K
0.35 × 0.09 × 0.03 mm

#### Data collection

Agilent Xcalibur with an Atlas (Gemini ultra Cu) detector diffractometer
Absorption correction: multi-scan (*CrysAlis PRO*; Agilent, 2010 ▶) *T*_min_ = 0.63, *T*_max_ = 1
14453 measured reflections
3221 independent reflections
3014 reflections with *I* > 3σ(*I*)
*R*_int_ = 0.034

#### Refinement

*R*[*F* ^2^ > 2σ(*F* ^2^)] = 0.023
*wR*(*F* ^2^) = 0.056
*S* = 1.37
3221 reflections
225 parameters
2 restraints
H atoms treated by a mixture of independent and constrained refinement
Δρ_max_ = 0.27 e Å^−3^
Δρ_min_ = −0.34 e Å^−3^
Absolute structure: Flack (1983 ▶), 1894 Friedel pairs
Flack parameter: 0.148 (19)

### 

Data collection: *CrysAlis PRO* (Agilent, 2010 ▶); cell refinement: *CrysAlis PRO*; data reduction: *CrysAlis PRO*; program(s) used to solve structure: *SIR2002* (Burla *et al.*, 2003 ▶); program(s) used to refine structure: *JANA2006* (Petříček *et al.*, 2006 ▶); molecular graphics: *DIAMOND* (Brandenburg & Putz, 2005 ▶); software used to prepare material for publication: *JANA2006*.

## Supplementary Material

Crystal structure: contains datablock(s) global, I. DOI: 10.1107/S1600536812001985/bx2397sup1.cif

Structure factors: contains datablock(s) I. DOI: 10.1107/S1600536812001985/bx2397Isup2.hkl

Supplementary material file. DOI: 10.1107/S1600536812001985/bx2397Isup3.cml

Additional supplementary materials:  crystallographic information; 3D view; checkCIF report

## Figures and Tables

**Table 1:** Hydrogen-bond geometry (Å, °) *Cg*2 is the centroid of the C6–C11 aromatic ring.

*D*—H⋯*A*	*D*—H	H⋯*A*	*D*⋯*A*	*D*—H⋯*A*
O1—H1*o*⋯N1	0.85 (3)	1.87 (3)	2.634 (3)	148 (3)
O2—H2*o*⋯N2	0.85 (3)	1.92 (3)	2.654 (3)	144 (3)
C11—H11⋯*Cg*2^i^	0.96	2.96	3.632 (2)	128

Symmetry code: (i) 
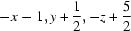
.

**Fig. 1.**
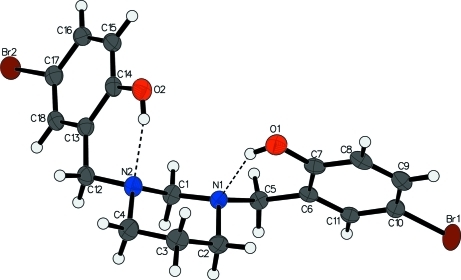
A view of the title molecule. Displacement ellipsoids are drawn at the 50% probability level. H atoms are drawn as small spheres of arbitrary radii and hydrogen bonds are indicated by dashed lines.

## supplementary crystallographic information

### Comment

In our group, research has been focused on the synthesis of *ortho*-Mannich bases and their structure, properties, and hydrogen-bonded properties. Very recently we reported the molecular structure of 2,2'-(dihydropyrimidine-1,3(2*H*, 4*H*)-diyldimethanediyl)bis-(6-tertbutyl-4-methoxyphenol) a novel di-Mannich base (Rivera, *et al.* 2012). Unlike related structure, the title compound crystallizes in an orthorhombic chiral *P*2_1_2_1_2_1_ space group. The molecular structure and atom-numbering scheme for (I) are shown in Fig. 1. In the crystal structure of the title compound (I), the hexahydropyrimidine ring adopts a chair conformation with a diequatorial substitution (Cremer & Pople, 1975) with puckering parameters Q, θ and φ of 0.591 (3) Å, 175.1 (3)°, 225 (3)°. The benzene rings makes an angle of 86.84 (10)° and 60.73 (10)° with the mean plane of heterocyclic ring defined by N1, C2, C4 and N2 atoms. The dihedral angle between the two benzene rings is 79.77 (10)°. In the molecule of the title compound , Fig. 1 bond lengths (Allen *et al.*, 1987) and angles are normal and comparable to the related structure (Rivera, *et al.* 2012).

There are two intramolecular hydrogen bonds between the phenolic hydroxy groups and nitrogen atoms with graph-set motif S(6) (Bernstein *et al.*, 1995). The shorter H—O distance [0.85 (3) Å] in comparison with the related structure (Rivera, *et al.* 2012), indicates a decreasing hydrogen-bonding strength, which is confirmed by the N···H and the N···O distances (Table 1). However, the observed C—O bond lengths [C7—O1 (1.355 (4) Å) and C14—O2 (1.365 (3) Å)] are shorter by 0.021 Å and 0.011 Å indicating a tendency to form a quinoid-type structure. Though, these C—O bond lengths are in good agreement with other related structure where the *p*-substituents in the aromatic ring is bromide [1.353 (2) Å] (Rivera, *et al.* 2011). We concluded that the bromine substituents do not induce considerably increase in hydrogen-bonding strength despite the contribution of this halogen atom in a quinoid structure by electron deslocalization.The crystal structure is stabilized by weak C—H···π interactions.

### Experimental

A solution of 1,3,7,9,13,15,19,21-octaazapentacyclo[19.3.1.1^3,7^.1^9,13^.1^15,19^]octacosane prepared according to a previous report (Rivera *et al.*, 2010) (200 mg, 0.54 mmol) in 96% ethanol (5 ml) was added slowly to a stirred solution of *p*-bromophenol (380 mg, 2.2 mmol) in 96% ethanol (5 ml) that was heated under reflux. Upon completion of the addition, the reaction mixture was stirred under reflux for 14 h. Next, the reflux was stopped, the solvent was removed on a rotary evaporator under vacuum, and the residue obtained was chromatographed on silica gel eluting with benzene/AcOEt (gradient elution with 5% to 20% AcOEt) to produce a solid which was recrystallized in 96% ethanol to provide high quality crystals of the title compound (I), (Yield 31.4%, m.p. 433–434 K)

### Refinement

The hydroxy hydrogen atoms were found in difference Fourier maps and their coordinates were refined with a distance restraint d(O—H) = 0.85 Å with σ 0.03 Å.

All other H atoms atoms were positioned geometrically and treated as riding on their parent atoms. The isotropic atomic displacement parameters of hydrogen atoms were evaluated as 1.2×*U*_eq_(C,O) of the parent atom .

### Crystal data

**Table:**

C_18_H_20_Br_2_N_2_O_2_	*F*(000) = 912
*M**_r_* = 456.2	*D*_x_ = 1.666 Mg m^−^^3^
Orthorhombic, *P*2_1_2_1_2_1_	Cu *K*α radiation, λ = 1.5418 Å
Hall symbol: P 2ac 2ab	Cell parameters from 8581 reflections
*a* = 5.9602 (3) Å	θ = 3.6–67.0°
*b* = 17.2164 (8) Å	µ = 5.76 mm^−^^1^
*c* = 17.7222 (8) Å	*T* = 120 K
*V* = 1818.53 (15) Å^3^	Plate, colourless
*Z* = 4	0.35 × 0.09 × 0.03 mm

### Data collection

**Table:**

Agilent Xcalibur with an Atlas (Gemini ultra Cu) detector diffractometer	3221 independent reflections
Radiation source: Enhance Ultra (Cu) X-ray Source	3014 reflections with *I* > 3σ(*I*)
mirror	*R*_int_ = 0.034
Detector resolution: 10.3784 pixels mm^-1^	θ_max_ = 67.2°, θ_min_ = 3.6°
Rotation method data acquisition using ω scans	*h* = −7→6
Absorption correction: multi-scan (CrysAlis PRO; Agilent, 2010)	*k* = −20→20
*T*_min_ = 0.63, *T*_max_ = 1	*l* = −21→20
14453 measured reflections	

### Refinement

**Table:**

Refinement on *F*^2^	Weighting scheme based on measured s.u.'s *w* = 1/(σ^2^(*I*) + 0.0004*I*^2^)
*R*[*F*^2^ > 2σ(*F*^2^)] = 0.023	(Δ/σ)_max_ = 0.009
*wR*(*F*^2^) = 0.056	Δρ_max_ = 0.27 e Å^−^^3^
*S* = 1.37	Δρ_min_ = −0.34 e Å^−^^3^
3221 reflections	Extinction correction: B-C type 1 Lorentzian isotropic (Becker & Coppens, 1974)
225 parameters	Extinction coefficient: 360 (70)
2 restraints	Absolute structure: Flack (1983), 1894 Friedel pairs
74 constraints	Flack parameter: 0.148 (19)
H atoms treated by a mixture of independent and constrained refinement	

### Special details

**Table:**

Experimental. CrysAlisPro (Agilent, 2010) Empirical absorption correction using spherical harmonics, implemented in SCALE3 ABSPACK scaling algorithm.
Refinement. The refinement was carried out against all reflections. The conventional *R*-factor is always based on *F*. The goodness of fit as well as the weighted *R*-factor are based on *F* and *F*^2^ for refinement carried out on *F* and *F*^2^, respectively. The threshold expression is used only for calculating *R*-factors *etc*. and it is not relevant to the choice of reflections for refinement.The program used for refinement, Jana2006, uses the weighting scheme based on the experimental expectations, see _refine_ls_weighting_details, that does not force *S* to be one. Therefore the values of *S* are usually larger than the ones from the *SHELX* program.

### Fractional atomic coordinates and isotropic or equivalent isotropic displacement parameters (Å^2^)

**Table:**

	*x*	*y*	*z*	*U*_iso_*/*U*_eq_	
Br1	0.51306 (8)	0.332316 (17)	1.176266 (16)	0.04042 (10)	
Br2	0.49477 (6)	0.037552 (13)	0.485209 (15)	0.03305 (8)	
O1	0.9595 (3)	0.38094 (11)	0.87498 (12)	0.0318 (6)	
O2	0.9653 (3)	0.28722 (12)	0.66366 (12)	0.0326 (6)	
N1	0.5696 (3)	0.35867 (12)	0.80850 (13)	0.0223 (6)	
N2	0.5782 (4)	0.36259 (13)	0.67419 (14)	0.0260 (7)	
C1	0.4884 (6)	0.32162 (12)	0.74006 (13)	0.0251 (7)	
C2	0.4815 (6)	0.43895 (13)	0.81379 (15)	0.0275 (7)	
C3	0.5591 (5)	0.48430 (15)	0.74549 (19)	0.0342 (10)	
C4	0.4950 (7)	0.44293 (13)	0.67271 (15)	0.0339 (8)	
C5	0.5110 (6)	0.31139 (12)	0.87501 (13)	0.0224 (6)	
C6	0.6347 (4)	0.33698 (14)	0.94497 (16)	0.0214 (7)	
C7	0.8526 (4)	0.36921 (14)	0.94151 (17)	0.0251 (8)	
C8	0.9639 (4)	0.38989 (13)	1.00746 (17)	0.0275 (8)	
C9	0.8650 (5)	0.37837 (14)	1.07713 (18)	0.0294 (9)	
C10	0.6495 (5)	0.34704 (14)	1.08040 (17)	0.0264 (8)	
C11	0.5363 (4)	0.32626 (13)	1.01568 (15)	0.0235 (7)	
C12	0.5203 (6)	0.32261 (13)	0.60354 (14)	0.0269 (7)	
C13	0.6347 (4)	0.24464 (16)	0.59565 (16)	0.0246 (8)	
C14	0.8500 (4)	0.23092 (16)	0.62571 (16)	0.0260 (8)	
C15	0.9503 (4)	0.15865 (16)	0.61723 (16)	0.0284 (8)	
C16	0.8460 (5)	0.10008 (17)	0.57645 (17)	0.0286 (8)	
C17	0.6373 (5)	0.11513 (16)	0.54497 (16)	0.0276 (8)	
C18	0.5310 (5)	0.18583 (14)	0.55486 (14)	0.0243 (7)	
H1a	0.327469	0.323535	0.73903	0.0301*	
H1b	0.537131	0.268477	0.73904	0.0301*	
H2a	0.537922	0.463141	0.858761	0.0329*	
H2b	0.320544	0.437631	0.814856	0.0329*	
H3a	0.492548	0.53507	0.746197	0.041*	
H3b	0.719001	0.490658	0.747452	0.041*	
H4a	0.334638	0.442663	0.667524	0.0406*	
H4b	0.559441	0.469861	0.63053	0.0406*	
H5a	0.544072	0.257805	0.864932	0.0269*	
H5b	0.352306	0.314336	0.883768	0.0269*	
H8	1.111007	0.412416	1.004589	0.033*	
H9	0.943604	0.39175	1.122599	0.0352*	
H11	0.388666	0.304252	1.01925	0.0282*	
H12a	0.559537	0.354959	0.561435	0.0322*	
H12b	0.360679	0.315774	0.60079	0.0322*	
H15	1.093883	0.149064	0.639922	0.0341*	
H16	0.916522	0.050365	0.570166	0.0343*	
H18	0.385015	0.194273	0.533472	0.0292*	
H1o	0.868 (5)	0.370 (2)	0.8398 (15)	0.0382*	
H2o	0.884 (5)	0.3266 (14)	0.672 (2)	0.0391*	

### Atomic displacement parameters (Å^2^)

**Table:**

	*U*^11^	*U*^22^	*U*^33^	*U*^12^	*U*^13^	*U*^23^
Br1	0.0547 (2)	0.04237 (16)	0.02419 (15)	−0.0043 (2)	0.00098 (18)	0.00131 (11)
Br2	0.04040 (16)	0.02472 (13)	0.03402 (15)	−0.00507 (16)	−0.00497 (17)	−0.00090 (10)
O1	0.0236 (11)	0.0369 (9)	0.0350 (11)	−0.0063 (8)	0.0030 (8)	0.0000 (8)
O2	0.0303 (11)	0.0359 (9)	0.0316 (10)	−0.0076 (9)	−0.0048 (9)	−0.0044 (8)
N1	0.0264 (12)	0.0177 (9)	0.0227 (11)	−0.0022 (7)	0.0022 (8)	0.0027 (9)
N2	0.0349 (12)	0.0187 (10)	0.0243 (12)	−0.0034 (8)	0.0010 (9)	0.0019 (9)
C1	0.0291 (13)	0.0211 (10)	0.0250 (12)	−0.0045 (14)	0.0021 (14)	0.0024 (9)
C2	0.0328 (13)	0.0193 (10)	0.0303 (13)	0.0024 (12)	0.0017 (15)	0.0003 (9)
C3	0.049 (2)	0.0172 (11)	0.0367 (17)	−0.0016 (11)	0.0032 (13)	0.0024 (11)
C4	0.0483 (15)	0.0235 (11)	0.0298 (13)	0.0023 (16)	0.0057 (19)	0.0069 (9)
C5	0.0221 (12)	0.0196 (9)	0.0256 (11)	−0.0050 (13)	0.0029 (14)	0.0017 (8)
C6	0.0202 (12)	0.0159 (11)	0.0281 (14)	−0.0005 (10)	−0.0020 (10)	0.0021 (10)
C7	0.0229 (13)	0.0173 (11)	0.0351 (16)	0.0026 (10)	0.0018 (11)	0.0034 (11)
C8	0.0233 (14)	0.0170 (10)	0.0421 (15)	−0.0023 (10)	−0.0081 (12)	0.0035 (10)
C9	0.0354 (15)	0.0160 (12)	0.0366 (17)	0.0013 (11)	−0.0112 (12)	−0.0026 (11)
C10	0.0363 (15)	0.0165 (12)	0.0265 (15)	0.0026 (11)	0.0004 (11)	0.0027 (10)
C11	0.0248 (14)	0.0174 (10)	0.0281 (13)	0.0036 (10)	−0.0006 (11)	0.0024 (9)
C12	0.0306 (14)	0.0264 (11)	0.0236 (12)	0.0002 (13)	−0.0007 (14)	0.0033 (9)
C13	0.0252 (13)	0.0278 (13)	0.0208 (13)	−0.0026 (10)	0.0011 (11)	0.0017 (11)
C14	0.0221 (13)	0.0337 (14)	0.0222 (14)	−0.0064 (11)	−0.0005 (10)	0.0021 (12)
C15	0.0244 (15)	0.0355 (13)	0.0254 (13)	0.0016 (11)	−0.0006 (10)	0.0023 (11)
C16	0.0305 (15)	0.0295 (14)	0.0259 (15)	0.0045 (11)	0.0012 (11)	0.0030 (12)
C17	0.0284 (14)	0.0296 (14)	0.0248 (14)	−0.0042 (11)	0.0028 (11)	0.0041 (12)
C18	0.0221 (14)	0.0291 (11)	0.0217 (11)	−0.0043 (11)	−0.0009 (11)	0.0052 (9)

### Geometric parameters (Å, °)

**Table:**

Br1—C10	1.901 (3)	C5—H5a	0.96
Br2—C17	1.905 (3)	C5—H5b	0.96
O1—C7	1.355 (4)	C6—C7	1.413 (4)
O1—H1o	0.85 (3)	C6—C11	1.396 (4)
O2—C14	1.365 (3)	C7—C8	1.390 (4)
O2—H2o	0.85 (3)	C8—C9	1.382 (4)
N1—C1	1.453 (3)	C8—H8	0.96
N1—C2	1.481 (3)	C9—C10	1.394 (4)
N1—C5	1.474 (3)	C9—H9	0.96
N2—C1	1.465 (3)	C10—C11	1.378 (4)
N2—C4	1.470 (3)	C11—H11	0.96
N2—C12	1.470 (3)	C12—C13	1.512 (4)
C1—H1a	0.96	C12—H12a	0.96
C1—H1b	0.96	C12—H12b	0.96
C2—C3	1.513 (4)	C13—C14	1.410 (4)
C2—H2a	0.96	C13—C18	1.389 (4)
C2—H2b	0.96	C14—C15	1.388 (4)
C3—C4	1.522 (4)	C15—C16	1.388 (4)
C3—H3a	0.96	C15—H15	0.96
C3—H3b	0.96	C16—C17	1.388 (4)
C4—H4a	0.96	C16—H16	0.96
C4—H4b	0.96	C17—C18	1.383 (4)
C5—C6	1.508 (4)	C18—H18	0.96
			
C7—O1—H1o	108 (2)	C7—C6—C11	118.5 (2)
C14—O2—H2o	111 (2)	O1—C7—C6	121.9 (3)
C1—N1—C2	110.1 (2)	O1—C7—C8	118.0 (2)
C1—N1—C5	110.24 (19)	C6—C7—C8	120.2 (3)
C2—N1—C5	112.4 (2)	C7—C8—C9	120.7 (3)
C1—N2—C4	110.1 (2)	C7—C8—H8	119.6439
C1—N2—C12	111.6 (2)	C9—C8—H8	119.6447
C4—N2—C12	110.3 (2)	C8—C9—C10	119.0 (3)
N1—C1—N2	109.4 (2)	C8—C9—H9	120.4851
N1—C1—H1a	109.4712	C10—C9—H9	120.4852
N1—C1—H1b	109.4714	Br1—C10—C9	118.9 (2)
N2—C1—H1a	109.4709	Br1—C10—C11	120.0 (2)
N2—C1—H1b	109.4715	C9—C10—C11	121.1 (3)
H1a—C1—H1b	109.5538	C6—C11—C10	120.5 (2)
N1—C2—C3	108.8 (2)	C6—C11—H11	119.773
N1—C2—H2a	109.4711	C10—C11—H11	119.7736
N1—C2—H2b	109.4713	N2—C12—C13	112.9 (2)
C3—C2—H2a	109.4717	N2—C12—H12a	109.4712
C3—C2—H2b	109.471	N2—C12—H12b	109.4714
H2a—C2—H2b	110.1093	C13—C12—H12a	109.471
C2—C3—C4	111.1 (2)	C13—C12—H12b	109.471
C2—C3—H3a	109.4713	H12a—C12—H12b	105.8417
C2—C3—H3b	109.4711	C12—C13—C14	121.6 (2)
C4—C3—H3a	109.4717	C12—C13—C18	119.6 (2)
C4—C3—H3b	109.4711	C14—C13—C18	118.7 (2)
H3a—C3—H3b	107.8053	O2—C14—C13	121.7 (2)
N2—C4—C3	109.9 (2)	O2—C14—C15	118.2 (2)
N2—C4—H4a	109.4721	C13—C14—C15	120.1 (2)
N2—C4—H4b	109.4708	C14—C15—C16	121.0 (2)
C3—C4—H4a	109.4712	C14—C15—H15	119.5034
C3—C4—H4b	109.4711	C16—C15—H15	119.5043
H4a—C4—H4b	109.0337	C15—C16—C17	118.4 (3)
N1—C5—C6	112.3 (2)	C15—C16—H16	120.817
N1—C5—H5a	109.4708	C17—C16—H16	120.8173
N1—C5—H5b	109.4703	Br2—C17—C16	119.5 (2)
C6—C5—H5a	109.4717	Br2—C17—C18	118.9 (2)
C6—C5—H5b	109.4716	C16—C17—C18	121.6 (3)
H5a—C5—H5b	106.4462	C13—C18—C17	120.2 (3)
C5—C6—C7	121.9 (3)	C13—C18—H18	119.8928
C5—C6—C11	119.6 (2)	C17—C18—H18	119.8919
			
?—?—?—?	?		

### Hydrogen-bond geometry (Å, °)

**Table:**

Cg2 is the centroid of the C6–C11 aromatic ring.

**Table:**

*D*—H···*A*	*D*—H	H···*A*	*D*···*A*	*D*—H···*A*
O1—H1o···N1	0.85 (3)	1.87 (3)	2.634 (3)	148 (3)
O2—H2o···N2	0.85 (3)	1.92 (3)	2.654 (3)	144 (3)
.—···..	.	.	.	.
C11—H11···Cg2^i^	0.96	2.96	3.632 (2)	128

Symmetry codes: (i) −*x*−1, *y*+1/2, −*z*+5/2.
